# Atezolizumab and paclitaxel as first line therapy in advanced triple-negative breast cancer patients included in the French early access program

**DOI:** 10.1038/s41598-023-40569-9

**Published:** 2023-08-18

**Authors:** Alexandre de Moura, Perrine Vuagnat, Benjamin Renouf, Jean-Yves Pierga, Delphine Loirat, Pauline Vaflard, Charline Lafayolle de la Bruyère, Natacha Chaumard-Billotey, Nawale Hajjaji, Sylvain Ladoire, Sandrine Dabakuyo, Anne Patsouris, Jean Sébastien Frenel, Vincent Nicolai, Marie Alexandre, Nadine Dohollou, Julien Grenier, Heloïse Bourien, François-Clément Bidard

**Affiliations:** 1 Department of Medical Oncology, Institut Curie, Paris & Saint Cloud, France; 2 UVSQ, Université Paris-Saclay, Saint Cloud, France; 3 Université Paris Cité, Paris, France; 4 Department of Medical Oncology, Centre Léon Bérard, Lyon, France; 5 Department of Clinical Pharmacy, Centre Léon Bérard, Lyon, France; 6 Department of Breast Cancer Oncology, Centre Oscar Lambret, Lille, France; 7 Inserm, U1192, Laboratoire Protéomique, Réponse Inflammatoire et Spectrométrie de Masse (PRISM), Univ. Lille, Lille, France; 8 Department of Medical Oncology, Centre Georges-François Leclerc, Dijon, France; 9 Department of Medical Oncology, Institut de Cancérologie de l’Ouest Pays de Loire, Angers & Nantes/Saint-Herblain, France; 10 Department of Medical Oncology, Institut Claudius Regaud-IUCT Oncopole, Toulouse, France; 11 Department of Medical Oncology, Institut du Cancer de Montpellier, Montpellier, France; 12 Department of Medical Oncology, Polyclinique Bordeaux Nord Aquitaine, Bordeaux, France; 13 Department of Medical Oncology, Institut Sainte-Catherine, Avignon, France; 14 Department of Medical Oncology, Centre Eugène Marquis, Rennes, France

**Keywords:** Cancer, Immunotherapy, Breast cancer

## Abstract

Following the results of the IMpassion130 trial, an early access program (EAP) was opened in France, allowing patients with PD-L1-positive advanced triple negative breast cancer (aTNBC) to receive a combination of paclitaxel and atezolizumab as first line therapy. This EAP was later discontinued when the IMpassion131 trial read out with negative results. We performed a retrospective multicentric analysis in patients who were prospectively enrolled in the French EAP. Efficacy and toxicity data were obtained on 64 patients treated from August 2019 to August 2020 in 10 French cancer centers. Median progression-free survival (PFS) and overall survival (OS) were 4.1 months (95% CI [3.0–5.8]) and 17.9 months (95% CI [12.4–NR]), respectively. The 6-months PFS rate was 28% (95% CI [16–40%]) (N = 18/64), while N = 33/64 patients (52%, 95% CI [38–63%]) experienced a tumor response. Exploratory subgroup analyses retrieved that corticosteroid use at inclusion in the EAP, before treatment initiation, was the only independent unfavorable prognostic factor for PFS (HR 2.7, 95% CI [1.3–5.6]). No new safety signal was observed. This real-life study, unique by its setting (EAP granted by anticipation and later withdrawn), suggests atezolizumab and paclitaxel has a limited efficacy in PD-L1-positive aTNBC, especially in patients receiving corticosteroids as comedication before treatment start.

## Introduction

Triple-negative breast cancer accounts for 15% of metastatic breast cancers, has a poorer prognosis and represents a high unmet medical need. There are biological arguments to support the use of immunotherapy in this tumor subtype, such as higher genomic instability, increased immune infiltration or higher level of programmed death-ligand 1 (PD-L1) expression^1,2^. Early clinical trials reported durable responses with the anti-PD-L1 monoclonal antibody atezolizumab as monotherapy or in association with nab-paclitaxel for advanced triple negative breast cancer (aTNBC)^3,4^. In the phase III IMpassion130 trial, atezolizumab with nab-paclitaxel in first line for aTNBC showed a statistically significant progression-free survival (PFS) benefit over nab-paclitaxel regardless of PD-L1 status, and a numerically longer overall survival (OS) in the PD-L1 positive tumor subgroup population^5–7^. Based on these results, atezolizumab has been the first immunotherapeutic agent to be approved for breast cancer by the U.S. Food and Drug Administration through an accelerated approval in March 2019. In France, an early access program (EAP) was also opened in August 2019 as first line therapy for PD-L1-positive aTNBC. However, since nab-paclitaxel is not reimbursed by the national health system, the EAP allowed patients to be treated with paclitaxel as chemotherapy backbone, although results on the paclitaxel-atezolizumab combination were still under investigation in the IMpassion131 phase III trial. One year later, in August 2020, the negative results of IMpassion131, which did not retrieve any benefit in PFS or OS from atezolizumab added to paclitaxel^8^, led to the discontinuation of the EAP. In this retrospective analysis of prospectively enrolled patients, we report the outcome of patients treated with atezolizumab and paclitaxel through the French EAP in major French breast cancer centers.

## Methods

### Patients and treatment

The French EAP was accessible to all patients and centers in France. Eligibility criteria were: patients aged 18 years old and more; aTNBC (estrogen and progesterone receptors < 10%, HER2-negative), by local assessment on the most recent tumor tissue available (i.e. patients presenting with a documented triple negative relapse of a previously treated non-triple negative primary tumor were eligible); no prior systemic treatment for aTNBC; PD-L1-positive (≥ 1%), using the Ventana PD-L1 SP142 assay and an immune cell score, which refers to the area occupied by PD-L1 positive immune cells as a percentage of the whole tumor area.

All patients treated as part of the EAP were prospectively registered at their site. They received a 1200 mg atezolizumab infusion every 21 days in addition to weekly paclitaxel, until progression, death, toxicity, or medical or patient decision.

### Data collection

This retrospective study was approved by the *Institut Curie* review board and was carried out in accordance with relevant guidelines and regulations. A synopsis of this study was shared with 28 French cancer centers, 10 of them had eligible patients and agreed to participate in this study. As part of the EAP, which is not a clinical trial, all patients were notified their pseudonymized data could be collected and analyzed, and how they can oppose to such use: patients who did not oppose to the use of their data could be included in this retrospective study. Written informed consent was deemed unnecessary by the *Institut Curie* review board in compliance with the European regulation n. 2016/679 about personal data protection.

Participating sites retrospectively collected data using electronic medical records for all patients included in the EAP. Data were collected regarding patients characteristics (birth date, sex, performance status), tumor characteristics (date of diagnosis, synchronous or metachronous metastases, tumoral characteristics at the localized stage and at the advanced stage, number and sites of metastases, PD-L1 expression, *BRCA* mutation status), medical history (previous neoadjuvant or adjuvant therapy, steroid use at baseline, defined as using at least 10 mg prednisone—or equivalent—daily for a week or more within 30 days before first atezolizumab injection), information on atezolizumab-paclitaxel prescription (start and end of treatment date, reason for discontinuation of treatment), safety (grade 3–5 toxicities according to CTCAE v4.0.1), treatment efficacy (with tumor assessment according to RECIST v1.1), and survival data. Data cutoff was January 21st, 2022. Pseudonymized individual data were manually reviewed for quality and coherence, and queries were issued whenever needed.

### Endpoints and statistics

This is a retrospective study of prospectively enrolled, consecutive patients. First endpoints were progression-free survival (PFS) and overall survival (OS). Because of its design, this study had no pre-specified power. Secondary endpoints were objective response rate (ORR, by RECIST criteria v1.1), exploration of prognostic and predictive factors, and safety. Given the small sample size, the purpose of subgroup analyses was purely exploratory, the following prognostic factors for PFS were investigated: age at baseline, performance status at baseline, previous chemotherapy, number and type of metastasis, PD-L1 expression, *BRCA* mutation, steroid use at baseline.

Descriptive statistics were used to summarize patient characteristics. Survival curves for PFS, median PFS and its 95% confidence interval (95% CI) were generated using the Kaplan–Meier method. Multivariate Cox proportional hazards models were constructed to identify independent prognosis factors. All factors significant at a conservative 5% level in univariate analysis were included in multivariate analysis. All analyses were performed using R version 3.3.2. Statistical significance was defined by a two-tailed p < 0.05.

### Ethical parameters

This retrospective study was approved by the *Institut Curie* review board, which is independent and not complied with any university, and was carried out in accordance with relevant guidelines and regulations. The study and its modality are registered as [AP-1TNBC—DATA200256].

## Results

### Patient characteristics

Individual data of 64 patients included in the atezolizumab EAP between August 2019 and August 2020 were contributed by 10 cancer centers. Data were collected until January 21st, 2022. Baseline demographic and clinicopathological characteristics of patients are shown in Table 1. All patients were women, with a median age of 56 years. Half of the patients, N = 33/64 (52%), had been previously treated with chemotherapy in neoadjuvant and/or adjuvant setting. Among the 59 patients with available data on the primary tumor phenotype, 10 (17%) had a non-triple negative primary breast cancer: ER-positive, N = 8/59 (14%), PR-positive, N = 4/59 (7%), HER2-positive, N = 2/59 (3%). All these patients were treated in the EAP following a proven triple negative relapse. PD-L1 expression was between 1 and 10% for N = 42/64 (66%) and was greater than 10% for N = 22/64 (34%). Brain metastases were present in N = 13/64 patients (20%). Steroid use at baseline was present in N = 10/64 patients (16%).

**Table 1:** Patient characteristics and prognostic factors.

Characteristic	N (%)	PFS (months) [95% CI]	HR [95% CI] univariate analysis	HR [95% CI] multivariate analysis
Female	64 (100%)			
Age				
> 55	31 (48%)	5.7 [3.0; 14.6]	1.00 (ref.)	1.00 (ref.)
≤ 55	33 (52%)	3.8 [2.7; 5.6]	1.45 [0.83; 2.53]	1.58 [0.78; 2.41]
Stage at initial diagnosis				
Stage I–II–III	18 (28%)	3.7 [2.7; 6]	1.00 (ref.)	
Stage IV (de novo metastatic disease)	46 (72%)	5.1 [3.5; NR]	0.75 [0.40; 1.41]	
No. of metastatic sites				
0	1 (2%)			
1	21 (33%)	4.0 [3.0; 8.0]	1.00 (ref.)	
2	19 (30%)	4.8 [3.0; NR]	0.79 [0.38; 1.61]	
≥ 3	23 (36%)	3.8 [2.5; 12.4]	0.98 [0.52; 1.87]	
Visceral metastasis				
No	27 (42%)	4.4 [3.0; 8.0]	1.00 (ref.)	
Yes	37 (58%)	4.1 [2.7; 6.0]	1.02 [0.58; 1.79]	
Bone metastasis				
No	32 (50%)	4.4 [3.5; 7.0]	1.00 (ref.)	
Yes	32 (50%)	3.8 [2.7; 14.6]	0.89 [0.51; 1.57]	
Lung metastasis				
No	43 (67%)	4.4 [3.0; 7.0]	1.00 (ref.)	
Yes	21 (33%)	4.1 [2.7; 14.6]	0.88 [0.49; 1.59]	
Liver metastasis				
No	46 (72%)	4.1 [3.2; 7.0]	1.00 (ref.)	
Yes	18 (28%)	4.3 [2.5; NR]	1.17 [0.64; 2.14]	
Brain metastasis				
No	51 (80%)	4.1 [3.0; 5.7]	1.00 (ref.)	
Yes	13 (20%)	5.6 [2.2; NR]	0.86 [0.43; 1.73]	
Initial tumor phenotype (in early stage)^a^				
Non-TNBC	10 (17%)	3.0 [2.7; NR]	1.00 (ref.)	
HR-positive (ER and/or PR ≥ 10%)	10 (17%)			
HER2-positive	2 (3%)			
TNBC	49 (83%)	4.1 [3.0; 5.7]	1.18 [0.55; 2.51]	
Performance status^a^				
0–1	52 (90%)	4.4 [3.2; 7.0]	1.00 (ref.)	
2–3	6 (10%)	6.1 [2.5; NR]	0.66 [0.24; 1.87]	
Prior chemotherapy				
No	31 (48%)	4.1 [3.2; 12.4]	1.00 (ref.)	
Yes	33 (52%)	3.7 [2.5; 8.0]	1.18 [0.68; 2.06]	
Prior taxane exposure				
No	33 (52%)	5.1 [3.7; 12.4]	1.00 (ref.)	
Yes	31 (48%)	3.0 [2.5; 7.5]	1.49 [0.86; 2.59]	
PD-L1 status				
1–10	42 (66%)	3.8 [3.0; 5.7]	1.00 (ref.)	
≥ 10	22 (34%)	4.8 [3.0; 17.3]	0.64 [0.34; 1.19]	
*BRCA* mutation status				
* BRCA* mutation not tested	29 (45%)			
* BRCA* mutation in tumor and/or germline	5 (8%)	3.8 [2.7; NR]	1.00 (ref.)	
No *BRCA* mutation found	30 (47%)	3.3 [2.7; 7.5]	0.86 [0.29; 2.53]	
Steroid use at baseline				
No	54 (84%)	4.8 [3.5; 8.0]	1.00 (ref.)	1.00 (ref.)
Yes	10 (16%)	2.6 [1.2; NR]	**2.86 [1.40**; **5.86]**	**2.73 [1.34**; **5.67]**

Significant values are in bold.

*HR* hormone receptor, *ER* estrogen receptor, *PR* progesterone receptor.

^a^Data on initial tumor phenotype and performance status were missing for N = 5 and N = 6 patients, respectively.

### Treatment efficacy and prognostic factors

**Figure 1:**
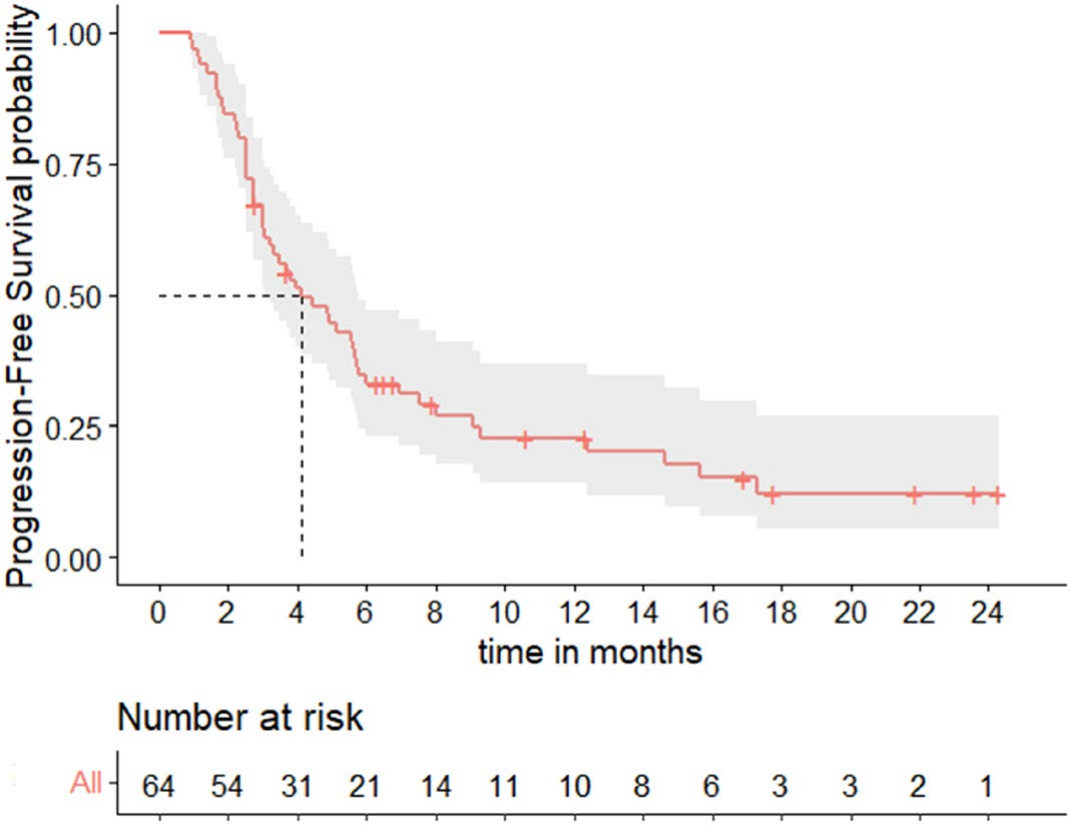
Progression-free survival.

**Figure 2:**
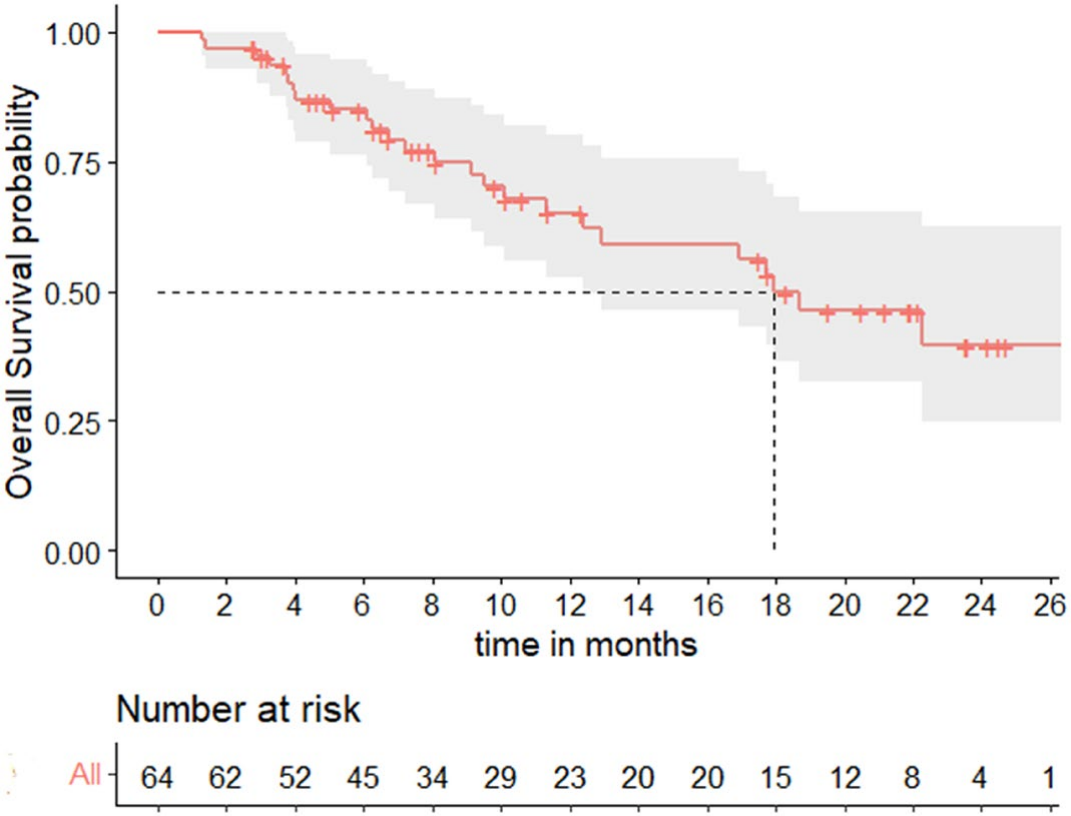
Overall survival.

In this cohort, median PFS was 4.1 months (95% CI [3.0–5.8]) (Fig. 1), with a 6-months PFS rate of 28% (95% CI [16–40%]). Median OS was 17.9 months (95% CI [12.4–NR]) (Fig. 2). The overall response rate (ORR) was 52% (95% CI [38–63%]): N = 6/64 patients (9%) had a complete response (CR) as best response, N = 27/64 (42%) had a partial response (PR), N = 4/64 (6%) had a stable disease (SD), and N = 27/64 (42%) had a progressive disease (PD).

**Figure 3:**
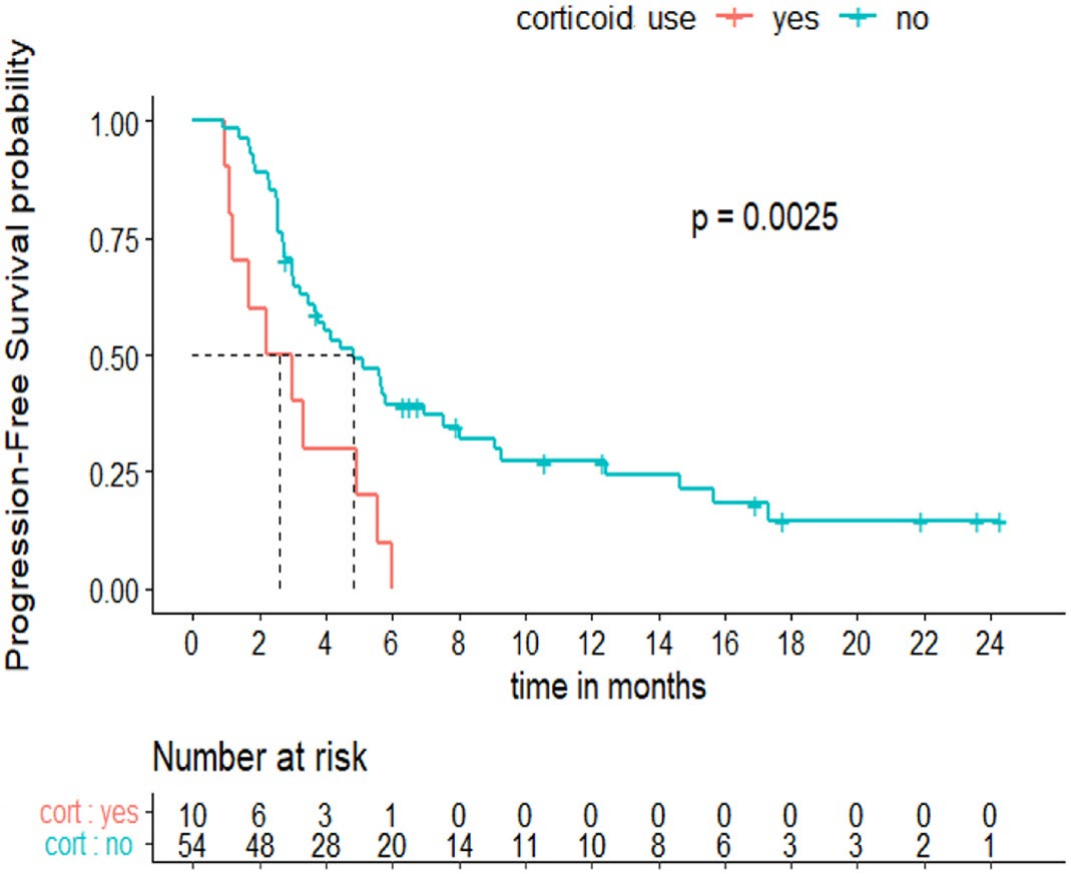
Progression-free survival according to steroid intake.

Univariate and multivariate analyses were undertaken to explore factors associated with PFS (Table 1). In multivariate analysis, the use of a steroid within one month before treatment initiation was the only independent adverse prognostic factor for PFS (HR 2.7, 95% CI [1.3–5.6], p = 0.0025) (Fig. 3). Other tested variables, such as age, initial tumor phenotype at early stage, disease type (recurrence versus de novo metastatic disease), the number of sites of metastatic disease, metastatic sites, previous treatment with chemotherapy, previous taxane exposure, PD-L1 level or *BRCA* mutation status had no significant impact.

### Safety

A toxicity of grade 3 or higher, according to CTCAE v4.0.1, was reported for 2 patients, with one case of grade 3 infusion related reaction, and one case of grade 3 myocarditis. There was no treatment-related death.

## Discussion

In the IMpassion131 trial, median PFS was 6.0 months with atezolizumab-paclitaxel versus 5.7 months with placebo-paclitaxel (HR 0.82, 95% CI [0.60–1.12]) whereas median OS was 22.1 months with atezolizumab-paclitaxel versus 28.3 months with placebo-paclitaxel (HR 1.11, 95% CI [0.76–1.64]), showing no difference between arms in the PD-L1-positive population. ORR was 63% with atezolizumab-paclitaxel and 55% with placebo-paclitaxel.

In our real-life cohort, median PFS was 4.1 months (95% CI [3.0–5.8]), median OS was 17.9 months (95% CI [12.4–NR]), and ORR was 52%. Such differences are often seen between clinical trials and real-life data since patients in clinical trials are often highly selected. In the present case, we can point out that a fifth of patients included in the French EAP had brain metastases. The actual percentage of patients with brain metastases in IMpassion131 was not reported but is expected to be low, since brain metastases had to be treated and controlled as stable prior to enrollment. Another potential difference in our population could be related to the proportion of patients whose primary breast cancers were not of triple negative phenotype. Noteworthy, neither pivotal trials for atezolizumab in aTNBC nor the atezolizumab label granted by FDA in 2019 (and later withdrawn) excluded these patients^5,8^. This phenotypic change was found in 17% of patients in our study and was not displayed in the IMpassion131 publication^5^. However, these data are in keeping with the ASCENT trial (investigating sacituzumab-govitecan in aTNBC patients), in which 30% of aTNBC patients had an initial HR-positive disease^9^.

An interesting finding of our analysis is the statistically significant association between initial corticosteroid use and shorter PFS. As pre-specified in our study protocol, prior corticosteroid use corresponded to an intake of at least 10 mg prednisone (or equivalent) daily for a week or more within 30 days before first atezolizumab injection. A limitation of this study is that we did not register the cause of such corticosteroid intake, observed in N = 10/64 patients (16%) of our cohort. On these 10 patients, 3 had brain metastasis, and two had low Performance Status, which can be confounding negative prognosis factors. However, 5 out of these 10 patients had no brain metastases, altered Performance Status, or multivisceral tumor invasion (defined as 3 or more metastatic sites) and corticosteroid use remained an independent factor of lower PFS in a multivariate model including these three criteria (results not shown). The design of our study prevents drawing any definitive explanation about the role of corticosteroids, which are often used in patients with symptomatic disease. Of note, the corticosteroid-induced immunosuppression could be also responsible for the observed impact on patient outcomes. Although a publication-based meta-analysis suggested that the concomitant administration of corticosteroids and immune checkpoint inhibitors may not necessarily lead to poorer clinical outcomes^10^, prospective studies revealed poorer outcomes with baseline steroid use at the initiation of anti-CTLA-4 therapy for melanoma^11,12^. In non-small-cell lung cancer, a negative impact of baseline steroids on efficacy of PD-1 and PD-L1 blockade has also been suggested^13^. If steroid use was responsible of a loss of activity of atezolizumab, we can also wonder about the effect of the weekly steroid premedication associated with paclitaxel. This could be one of the hypotheses to explain why atezolizumab improved the patient outcomes in IMpassion130 study with nab-paclitaxel (no steroid premedication needed) but not in IMpassion131 study with paclitaxel^14^. Of note, in the KEYNOTE-355 study, the anti-PD1 therapy pembrolizumab in association with first-line chemotherapy for aTNBC was associated with a benefit in PFS and OS, whatever the chemotherapy backbone^15^, suggesting no impact of steroid premedication on pembrolizumab efficacy although this study was not designed to compare outcomes according to the chemotherapy backbone. Another hypothesis that could explain differences between paclitaxel-atezolizumab and nab-paclitaxel-atezolizumab in the IMpassion130 and IMpassion131 studies is a difference in study populations. Although the clinical criteria are similar in the two studies and between the two arms of each study (disease stage and metastatic sites, previous treatments, median age, performance status, PD-L1 expression), the populations may differ on tumor biological criteria influencing prognosis and treatment efficacy other than PDL1 expression and not reported, such as tumor-infiltrating lymphocytes (TILs) expression or tumor mutation burden (TMB) which may be unbalanced. The surprisingly high median overall survival in the standard arm of IMpassion131 could be indicative of a population with a better prognosis and support this hypothesis^14^.

In addition to the IMpassion trials, another immuno-chemotherapy combination has recently been reported: ALICE was a first-line aTNBC blinded randomized phase II trial which evaluated the addition of atezolizumab to a combination of pegylated liposomal doxorubicin and low-dose cyclophosphamide. This backbone chemotherapy regimen was based on the perceived immunogenic properties of anthracyclines, the avoidance of steroids and the reported effects of low-dose cyclophosphamide on regulatory T cells. This study reported a PFS benefit with atezolizumab, with an increase in long term responders^16^. These findings support the hypothesis that the type of chemotherapy combined with immunotherapy has a role in triggering the immune response in aTNBC.

While pivotal randomized trials are intended to formally demonstrate the efficacy of a new treatment (or lack of thereof), the “real-world” represent an important supplementary source of post-approval clinical data. A good example is our finding related to the association between corticosteroid use and PFS; such association could not be reported in the original trial, since patients under corticosteroids were excluded from IMpassion130 study. Of note, the framework of our study, which included consecutive patients treated in multiple centers as part of the French early access program, make our report more robust than most real-world evidence reports based on purely retrospective analyses. However, the main limitation of our study is the small sample size which precludes any definitive conclusions, particularly with regard to subgroup analyses.

Finally, although no new or unexpected serious adverse event has been observed in this cohort, our report suggests that paclitaxel and atezolizumab has little benefit as first line therapy for PD-L1-positive aTNBC, especially in patients receiving corticosteroids as comedication before treatment start.

## Data Availability

The datasets generated during and/or analyzed during the current study are available from the corresponding author on reasonable request.
